# Insights on metallic particle bonding to thermoplastic polymeric substrates during cold spray

**DOI:** 10.1038/s41598-022-22200-5

**Published:** 2022-10-27

**Authors:** Asghar Heydari Astaraee, Chiara Colombo, Sara Bagherifard

**Affiliations:** Department of Mechanical Engineering, Politecnico di Milano, 20156 Milan, Italy

**Keywords:** Mechanical engineering, Structural materials

## Abstract

Metallization of polymers using cold spray technology has reached wide consideration in recent years. However, an effective modeling approach to address the deposition phenomena able to assess bonding formation in polymer metallization is still lacking. This study aims to develop a finite element model to simulate the solid-state deposition of metallic particles on thermoplastic polymeric substrates. Single copper particle impact on the Polyether Ether Ketone substrate was modeled using the coupled Lagrangian–Eulerian approach. Emphasis was given to the polymer material properties and substrate thermal history to account for the sensitivity of the physical and mechanical properties of polymers to temperature. Experimental coating depositions were performed to select an optimized set of spray parameters while single-particle impact tests were conducted for model validation. The substrate temperature was measured using an infrared thermal camera and was used to model the sub-surface temperature gradient during gas spray exposure. The proposed numerical model is shown to be capable of predicting various impact features includi mechanical interlocking and the effect of particle velocity fluctuations and temperature gradients on the extent of bonding. Substrate heating was found to have a distinct effect on the correct prediction of particle bonding. The proposed model enables tuning the appropriate processing conditions for successful copper particle adhesion on PEEK polymeric substrates.

## Introduction

Polymers and polymeric composites are efficient materials desired for lightweight construction. However, their application is limited due to low electrical and thermal conductivity, and not sufficient wear resistance. Hence, an emerging demand exists for combining polymers with metals, which is commonly referred to as polymer metallization to address these shortcomings. Thermal spray is known as an efficient polymer metalizing method in terms of high deposition rates, low costs, and industrialization^1^. Among thermal spray methods, cold spray stands out for the lower working temperatures that minimizes if not hinders the degradation of the polymeric substrate and melting/oxidation problems^2,3^.

The success of cold spray deposition on polymers depends on the development of the first layer on the substrate since substrate erosion almost always competes with coating deposition^4^. The erosion problem is mostly reported for epoxy resin composites^5^ and is less encountered in thermoplastic substrates^6^. Relatively uniform solid-state metallic coatings have been deposited on thermoplastics such a polyamide^7^, polyether ether ketone (PEEK)^8–10^, carbon-fiber reinforced PEEK^11,12^ and polycarbonate^13,14^. In addition to erosion, thermal degradation of the substrate is inevitable at high processing temperatures^15^. These conditions create limitations on the available deposition window and consequently, the choice of processing parameters (such as gas pressure and temperature) becomes critical.

First layer deposition is also important for the structural integrity and the adhesion strength of the coating^16^. It is obvious that service conditions will impart certain limitations on the required minimum adhesion strength of structural metalized polymers and composites. An ongoing challenge with the solid-state cold spray coatings on polymers and composites is the relatively low adhesion strength of deposited layers, reported to be in the range of 5–10 MPa^14,17–19^. Considering various structural thermoplastic polymers, the generic design stress is a proportion (1/2–2/3rd) of the yield strength which is higher than the obtained adhesion strengths. Only very few studies have reported adequate adhesion strengths for specific powder/polymer combinations, corresponding the to substrate material used^20^. A fact that should be addressed here is that due to the relatively distinct nature of metals and polymers, obtaining a strong chemical bond between them is tricky. That is why it is generally accepted that the main bonding mechanism in action for the solid-state metallic coatings on polymers is mechanical interlocking^21–23^. Thus, in the current study and throughout this paper, mechanical interlocking and anchorage are meant as “particle bonding”.

Analyzing the available literature indicated that there is limited knowledge on the bond formation and its governing parameters. Consequently, understanding particle interlocking with the polymeric substrates and the acting parameters is crucial for the selection of cold spray parameters to produce coatings with high deposition efficiency and sufficient adhesion strength. In this context, simulations are intended to develop efficient models for predicting particle deformation and bonding phenomena. In a recent study by the authors^24^, analyzing the state of the art indicated a lack of efficient numerical models in the literature for appropriately describing the related bonding phenomena. Thus, a numerical finite element (FE) model was developed for simulating the metallization of PEEK thermoplastic polymer using cold spray deposition based on the impact of a single particle. It is noted that the development of a single particle model is a primary step for investigating the bonding mechanism and thus more efficient multi-particle models. The substrate was modeled using an Eulerian formulation that eliminates mesh distortion at large deformations and hence the need for inputting a material damage model. The PEEK material model was calibrated using high strain rate data to catch a more realistic dynamic deformation behavior. The model enabled the prediction of the deformation behaviors of the particle/substrate and whether the particle remained embedded or rebounded after the impact depending on its kinetic energy. The proposed model was shown to be a useful tool for the cold spray parameter selection and predicting the required particle velocity for successful particle interlocking. Very similar to the concept of critical velocity as a threshold for metallurgical bonding in the cold spray of metals on metals, a minimum velocity called anchorage or interlocking velocity can be defined below which no effective particle embedment occurs. for a detailed discussion, the reader is referred to^24^. Concurrently, Tsai et al.^25^ developed a numerical Lagrangian model of single-particle impact in the cold spray process for Cu and alumina powder and polyamide substrate combinations. Differently, they used a three-network material model for the constitutive behavior of the polymer and applied simple failure criteria to hinder mesh distortion problems of the Lagrangian domain. In agreement with our previous model, they also showed that a minimum particle velocity or kinetic energy is required to hinder the particle detachment from the substrate and embed it into the polymer.

In the current study, an extension of our previous model^24^ is proposed, implementing further developments. In particular, in the new model, we have used a more realistic evolution of material properties of the polymeric substrate and also considered the effect of substrate temperature gradient on particle bonding phenomena. In addition, a complementary development is suggested to consider the effect of the gas stream as a contributing factor to promote particle interlocking. Single-particle impact tests, as well as coating deposition experiments, were performed for model validation purposes. These modifications resulted in significant developments with respect to the previous model. The results confirmed that the proposed model can predict the impact features matching the ones observed in single-particle impact and full coating deposition tests. The extent and variation of the impact features contributing to bonding are discussed using the proposed model.

## Experimental tests

### Cold spray deposition

**Figure 1:**
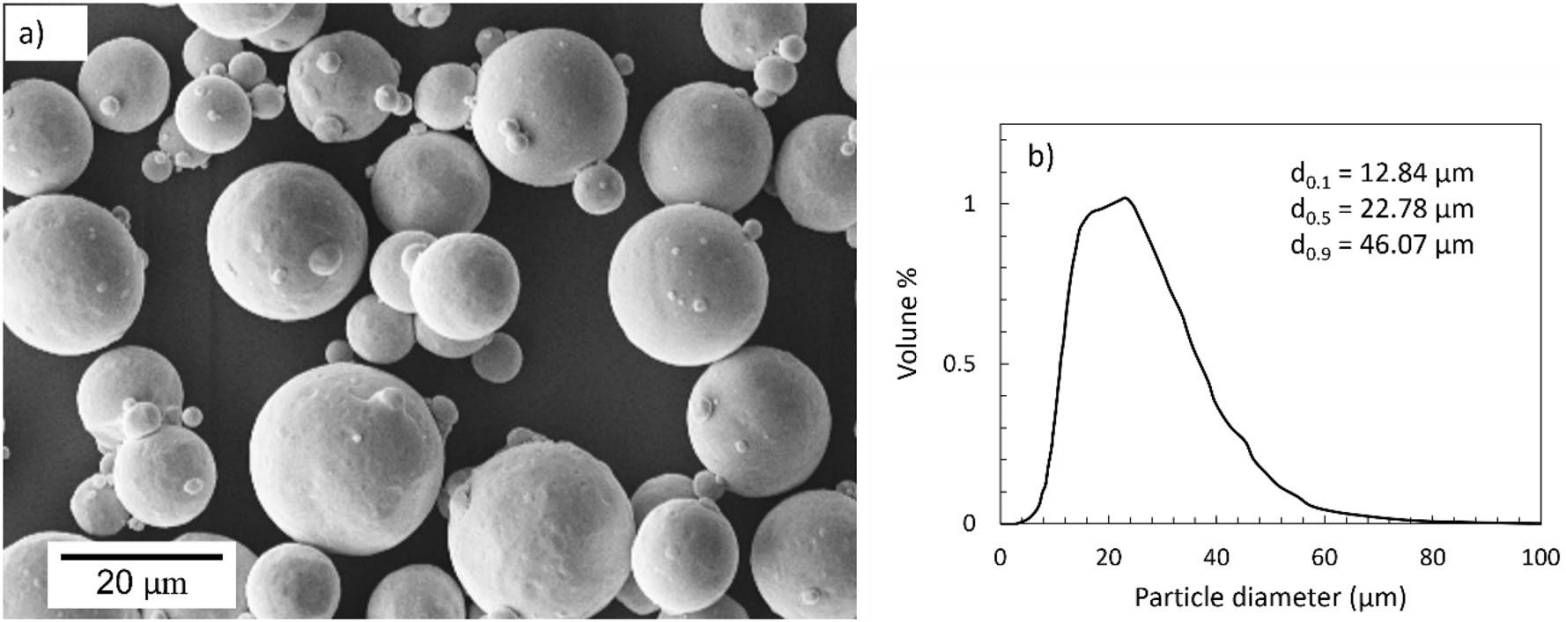
SEM micrograph (**a**) and size distribution (**b**) of copper powder feedstock.

Cold spray deposition was carried out using $5/8$ Impact high-pressure cold spray system (Impact Innovations, DE) equipped with an OUT1 de Laval converging-diverging nozzle (Impact Innovations, DE) with a length of 160 mm and an expansion ratio of 5.6. The movement of the cold spray gun was controlled by a robot (KUKA, GmbH, DE). Planar PEEK (RS Components Srl., Italy) with dimensions of $40 \times 45 \;{\text{mm}}^{2}$ in the as-received surface condition was used as the substrate material. Gas atomized oxygen-free copper powder feedstock ($>99.5\%,$ Safina, as, CZ) with spherical particles and an average particle size of 22.78 µm (d_0.1_ = 12.84 µm, d_0.5_ = 22.78 µm, d_0.9_ = 46.07 µm) measured using a Malvern Morphologi 4 particle size analyzer (Malvern Panalytical Ltd., UK) was used as shown in Fig. 1. The substrate surface was degreased with ethyl alcohol before deposition. Table 1 shows the cold spray parameters both for single-impact tests and coating deposition. Samples were fixed at a stand-off distance (SoD) equal to $40 \;{\text{mm}}$ from the nozzle. A larger SoD is preferred for the cold spray on polymers using high-pressure systems to moderate thermal exposure of the substrate^4,19^. Nitrogen was used as the process and carrier gases. The feeder was charged in a way that a fully covered conveyor disk by the powder was always ensured. Several trials were done to tune the processing parameters to obtain a homogeneous and apparently uniform coating. Thus, gas pressure and temperature were set to $4\; {\text{MPa}}$ and $300 \;^\circ {\text{C}}$, respectively. To obtain single-particle impacts, the powder feed rate was lowered drastically while the nozzle traverse speed was considerably increased compared to the setting used for full coverage coating deposition, as listed in Table 1. A scanning step of $1.0 \;{\text{mm}}$ was set for the deposition of full coating.

**Table 1:** Cold spray processing parameters for single-particle impact (wipe-test) and coating build-up experiments.

Test type	SoD (mm)	Feed rate (g/min)	Nozzle traverse speed (mm/s)	Gas pressure (MPa)	Gas temperature ($^\circ \mathrm{C}$)
Wipe-test	40	1.5	700	4	300
Coating deposition	40	25	30	4	300

### Temperature measurement

To estimate the thermal history of the substrate during single-particle impact deposition, temperature measurements were performed using a high-speed infrared camera (FLIR X6900sc, Teledyne FLIR, LLC, USA). The camera was placed with an angle equal to 45 degrees with respect to the specimen surface and at a distance of about $40 \;{\text{cm}}$. The surface temperature of the polymer was recorded during cold spraying with a scanning step of $1.0 \;{\text{mm}}$ for the case of coating build-up pattern. The powder feeding was not used during these measurements, to reduce the contamination risks of the camera, and thus, the sapphire protective window was removed during the recording. Therefore, in these experiments only the flowing gas contributed to the thermal history of the substrate. This would be a logical assumption for the single-particle impact experiments where there is no effective coating buildup during the spray. Thermal calibration was automatically performed before spraying. The acquisition frequency was set to 1920 Hz with a window of $290\times 200$ pixel, to store the temperature history with sufficient time and space resolution. The recoded data were processed with the software Research IR v. 4.40.11 (Teledyne FLIR, LLC, USA)^26^. The emissivity of the PEEK was considered a constant value of 0.95 as reported by Hirschen and Gülhan^27^ from room temperature to the melting point. A sensitivity analysis was performed to confirm the stability of the peak temperature as a function of slight changes in the emissivity value.

### Sample characterization

The sprayed samples were studied using an Leitz Aristomet optical microscope (OM) and a Zeiss EVO50 scanning electron microscope (SEM). To examine the single-particle impacts, the samples were coated with gold using a sputter coater (Agar Scientific, Ltd., UK) to render high-quality images. To analyze the cross-sections of the coatings, the samples were cut using a diamond saw and were grinded using abrasive papers up to $P2000$ and then mechanically polished using $0.3 \upmu \mathrm{ m}$ diamond paste.

## Numerical modeling

### FE single-particle impact model

Modeling and simulation procedures were fulfilled using the FE software Abaqus/Explicit 2019 (Dassault Systèmes SE, France)^28^. The numerical model definition including the model geometry is based on the authors’ previous work^24^. Thus, in this section, only the most important aspects and modifications with respect to the previous work are addressed. A Lagrangian domain was adopted for the Cu particle since it does not undergo intense deformations during the impact on the softer polymeric substrate. Instead, the substrate was modeled using Eulerian domain that allows the flow of the material through a fixed mesh in space, and thus, avoids conventional mesh distortion problems as a result of severe deformation. However, Eulerian domains require three-dimensional (3D) modeling. Hence, to lower the computational cost, a quarter of a full model was considered with symmetric boundary conditions. The spherical particle had a representative size equal to 20 µm while the substrate was considered relatively larger (length and height equal to 100 µm) to reduce the effects of boundary conditions on the interested zone. A coupled structural-thermal analysis was used to build up a more accurate model of the thermal analysis for the consideration of after-impact phenomena including particle rebound. Therefore, the particle and the substrate were meshed using respectively 15,040 and 254,889 solid elements containing 8 nodes with hourglass control and reduced integration. In the critical regions with high deformations, a fine mesh size was used (~ 1/45th particle size obtained by mesh convergence analysis) to increase the accuracy of the simulations while maintaining an affordable computational cost.The displacement and rotational degrees of freedom of the bottom surface were fixed to confine the substrate. The particle impact direction was considered perpendicular to the initial surface. Particle velocity (${V}_{p}$) and temperature (${T}_{p}$) were obtained from Kinetic Spray Solutions (KSS) software (v. 1.4.2) using the experimental single-particle impact test parameters and incorporated as initial conditions. KSS software relies on computational fluid dynamic (CFD) simulations of the two-phase flow to estimate particle impact conditions in terms of temperature and velocity. Details on the KSS basis and its calculation procedures are available in^29,30^. For the initial substrate temperature (${T}_{s}$), different conditions of either gradient or uniform distribution were applied based on the thermal domain induced by nozzle traverse speed.

Contact between the particle and the substrate was modeled by assuming a constant friction coefficient equal to 0.35^31,32^ adopting a general contact algorithm. Heating induced by friction and plastic deformation was considered in the model, considering an inelastic heat fraction of 0.9 and the default settings for heat generation in Abaqus. Material properties and the corresponding constants for copper and PEEK are listed in Table 2^33,34^. As regards the PEEK, there are two material property sets in the table. The original set is the one used in a previous work by authors^24^, while the modified set is the improved one that is proposed in the current work regarding the elastic-plastic behavior of PEEK and its sensitivity to temperature. Young’s modulus of PEEK rises with increasing plastic strain and strain rate and decreases with temperature; thus, two representative values of 10 GPa for room temperature and 4 GPa for $200 \;^\circ {\text{C}}$ were adopted from the literature that fit the dynamic behavior more suitably^35^. As regards the plastic deformation behavior, the material constitutive for the particle and the substrate was modeled using the Johnson–Cook plasticity relation that considers the effects of strain rate and temperature:

1 $$\sigma =\left[A+B{\varepsilon }_{p}^{n}\right]\left[1+C ln\left(\frac{\dot{\varepsilon }}{\dot{{\varepsilon }_{0}}}\right)\right]\left[1-{(\frac{T-{T}_{0}}{{T}_{m}-{T}_{0}})}^{m}\right]$$

where $\sigma$, ${\varepsilon }_{p}$, $\dot{\varepsilon }$ and $T$ are the flow stress, plastic strain, plastic strain rate, and temperature, respectively. $A$, $B$, $n$, $C$, $m$, ${\dot{\varepsilon }}_{0}$, ${T}_{0}$ and ${T}_{m}$ are material constants. Although the Johnson–Cook model is not fully able to capture the viscoplasticity of PEEK, it was the only available immediate approximation regarding the complexities of characterizing the viscoplastic behavior of PEEK at extremely high strain rates and temperatures encountered in CS. In the modified model, compressive stress–strain curves from the literature^36^ were used for a better calibration of the hardening constant and exponent ($B$, $n$). Accordingly, the parameters $C$, $m$, $B$, and $n$ were recalibrated at dynamic strain rates by fitting the material constitutive law to the experimental data using the least-squares method. It is noted that PEEK, like many thermoplastic polymers, has almost no mechanical resistance above a certain temperature and becomes a viscous fluid. This is the reason behind the calibration of ${T}_{m}$. The value of ${T}_{m}$ was obtained by extrapolating the compression yield stress data as a function of temperature to zero stress (see Fig. S1). The material constitutive model was implemented through a VUHARD subroutine.

**Table 2:** Physical and mechanical constants of copper and PEEK.

Property	Copper	PEEK (original)	PEEK (modified)
Density ($\mathrm{kg}/{\mathrm{m}}^{3}$)	8960	1300	1300
Young’s modulus ($\mathrm{MPa}$)	124	3500	4000 ($T=200\;^\circ \mathrm{C}$) 10,000 ($T=25\;^\circ \mathrm{C}$)
Poisson's ratio	0.34	0.40	0.40
Thermal conductivity ($\mathrm{W}/\mathrm{m}. ^\circ \mathrm{C}$)	386	0.25	0.25
Specific heat ($\mathrm{J}/\mathrm{kg}. ^\circ \mathrm{C}$)	383	Variable, see text	Variable, see text
Elastic limit, $A$ ($\mathrm{MPa}$)	90	132	132
Hardening constant, $B$ ($\mathrm{MPa}$)	292	10	32.5
Hardening exponent,$n$	0.31	1.2	3.5
Strain rate constant, $C$	0.025	0.029 ($\dot{\varepsilon } < 100{ }\;{\text{s}}^{ - 1}$)	0.0278 ($\dot{\varepsilon } < 20\;{\text{s}}^{ - 1}$)
		0.0834 ($\dot{\varepsilon } \ge 100{ }\;{\text{s}}^{ - 1}$)	0.1373 ($\dot{\varepsilon } \ge 20\;{\text{s}}^{ - 1}$)
Reference strain rate, $\dot{\varepsilon }_{0}$ $({\text{s}}^{ - 1}$)	1.0	0.001 ($\dot{\varepsilon } < 100\;{\text{ s}}^{ - 1}$)	0.001 ($\dot{\varepsilon } < 20{ }\;{\text{s}}^{ - 1}$)
		1.0 ($\dot{\varepsilon } \ge 100{ }\;{\text{s}}^{ - 1}$)	1.0 ($\dot{\varepsilon } \ge 20\;{\text{ s}}^{ - 1}$)
Thermal exponent,$m$	1.09	0.634	1.123 ($\dot{\varepsilon } < 20\;{\text{ s}}^{ - 1}$) 2.01 ($\dot{\varepsilon } \ge 20{ }\;{\text{s}}^{ - 1}$)
Melting temperature, ${T}_{m}$ ($^\circ \mathrm{C}$)	1083	341	251
Reference temperature, ${T}_{0}$ ($^\circ \mathrm{C}$)	25	23	23
Inelastic heat fraction	0.9	0.9	0.9

### Transient thermal analysis

**Figure 2:**
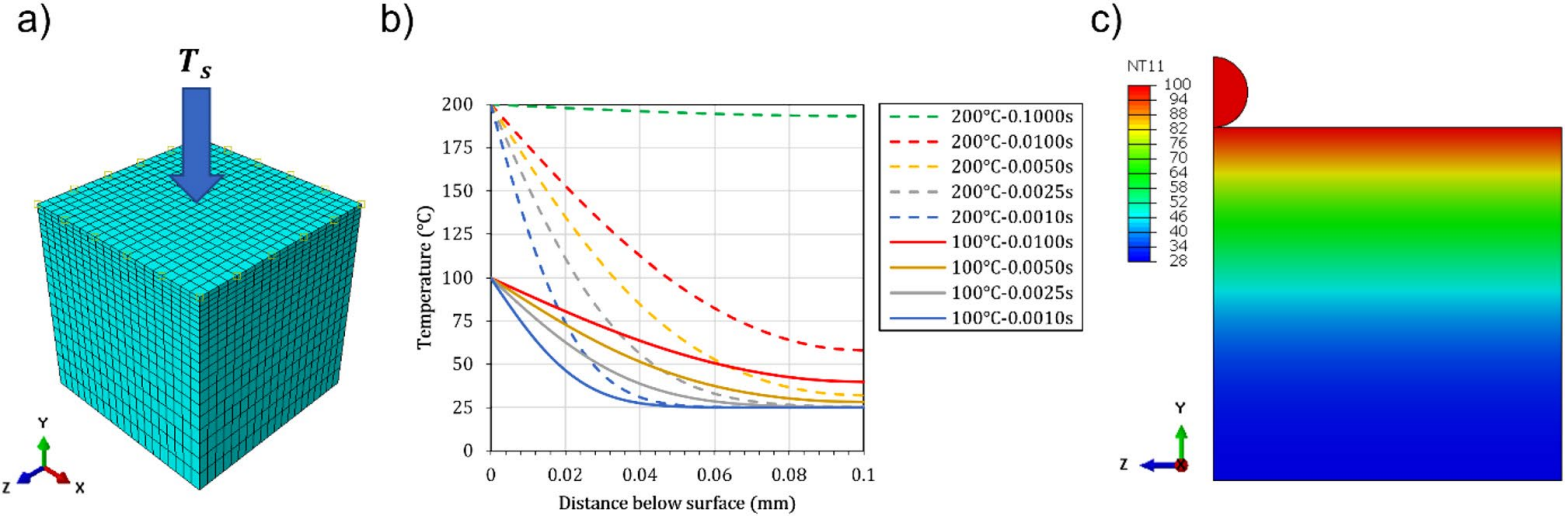
(**a**) Thermal analysis FE model for the estimation of the temperature gradient in the topmost surface (**b**) typical superficial temperature gradients obtained as an outcome of the thermal model (**c**) application of thermal gradients as an initial condition to the single-particle impact model.

Another FE model was developed to estimate the temperature gradient in the topmost surface layer of the substrate during deposition. The FE model was built-up using Abaqus/Standard 2019 (Dassault Systèmes SE, France), as shown in Fig. 2a. A cubic domain with an edge dimension equal to 0.1 mm was meshed using heat transfer elements with an element size of 0.005 mm. The upper surface temperature (${T}_{s}^{u}$) was fixed at the desired one (100 and 200 $^\circ \mathrm{C}$) measured by the thermal camera corresponding to the relevant nozzle speed (700 and 30 $\mathrm{mm}/\mathrm{s}$). Then a transient thermal analysis was performed with the required step time matching the exposure time ${(t}_{e})$ of any arbitrary point over the surface. Table 2 lists the exposure times for some typical nozzle traverse speeds for a flow spot diameter equal to 8 mm considering a constant nozzle velocity. The outcome of this FE simulation, which is the temperature gradient through the depth (as shown in Fig. 2b), was applied as an initial condition to the single-particle impact FE model (Fig. 2c). It is worth mentioning that the typical spray intervals reported in Table 2 (which are above several milliseconds) are much longer than the periods encountered during particle impacts (normally in the order of nanoseconds).

## Results

### Superficial substrate temperature

**Figure 3:**
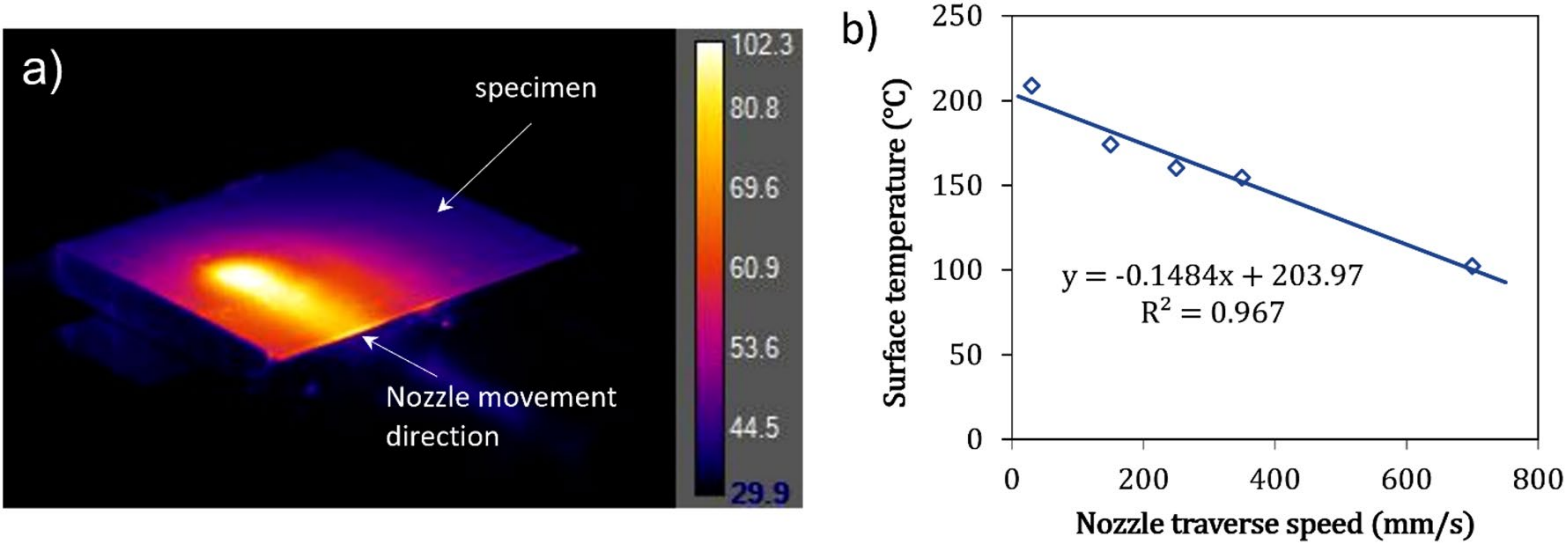
(**a**) Contour of temperature distribution (°C) during cold spraying the PEEK substrate with a linear scan pattern and a scanning step of $1\; \mathrm{mm}$ at a nozzle speed of $0.7\;\mathrm{ m}/\mathrm{s}$ with no powder (**b**) induced surface temperature over the spray spot as a function of nozzle traverse speed.

A typical temperature contour recorded by the thermal camera during spraying corresponding to the time when the nozzle is over the specimen is shown in Fig. 3a. The nozzle movement track is also indicated by the arrow. The maximum induced temperature by the moving gas flow was recorded for different nozzle traverse speeds in separate trials. It was found that the maximum temperature under the flow spot is almost constant during the spray. On the other hand, Fig. 3b shows the experimentally measured maximum temperature as a function of the nozzle traverse speed. A linear trend was observed between the nozzle speed and the maximum induced surface temperature. As the nozzle traverse speed decreases, the surface temperature increases due to longer exposure time and more effective convective heat flow. For this particular set of spray parameters (gas pressure of 4 MPa and gas temperature of $300\; ^\circ {\text{C}}$), the maximum surface temperature of around $200\; ^\circ {\text{C}}$ was induced at very low nozzle traverse speeds. At very long exposure times, it is anticipated that the surface temperature may match the gas flow temperature immediately on the surface. This is fully in agreement with the results obtained by the computational fluid dynamic (CFD) analysis performed using KSS software based on the same experimental process parameters (Nozzle OUT1, SoD of $40{ }\;{\text{mm}}$, gas pressure of $4\;{\text{ MPa}}$, gas temperature of $300 \;^\circ {\text{C}}$), which estimated a gas temperature of $203\;{ }^\circ {\text{C}}$ at the substrate level.

### Single-particle impact test

**Figure 4:**
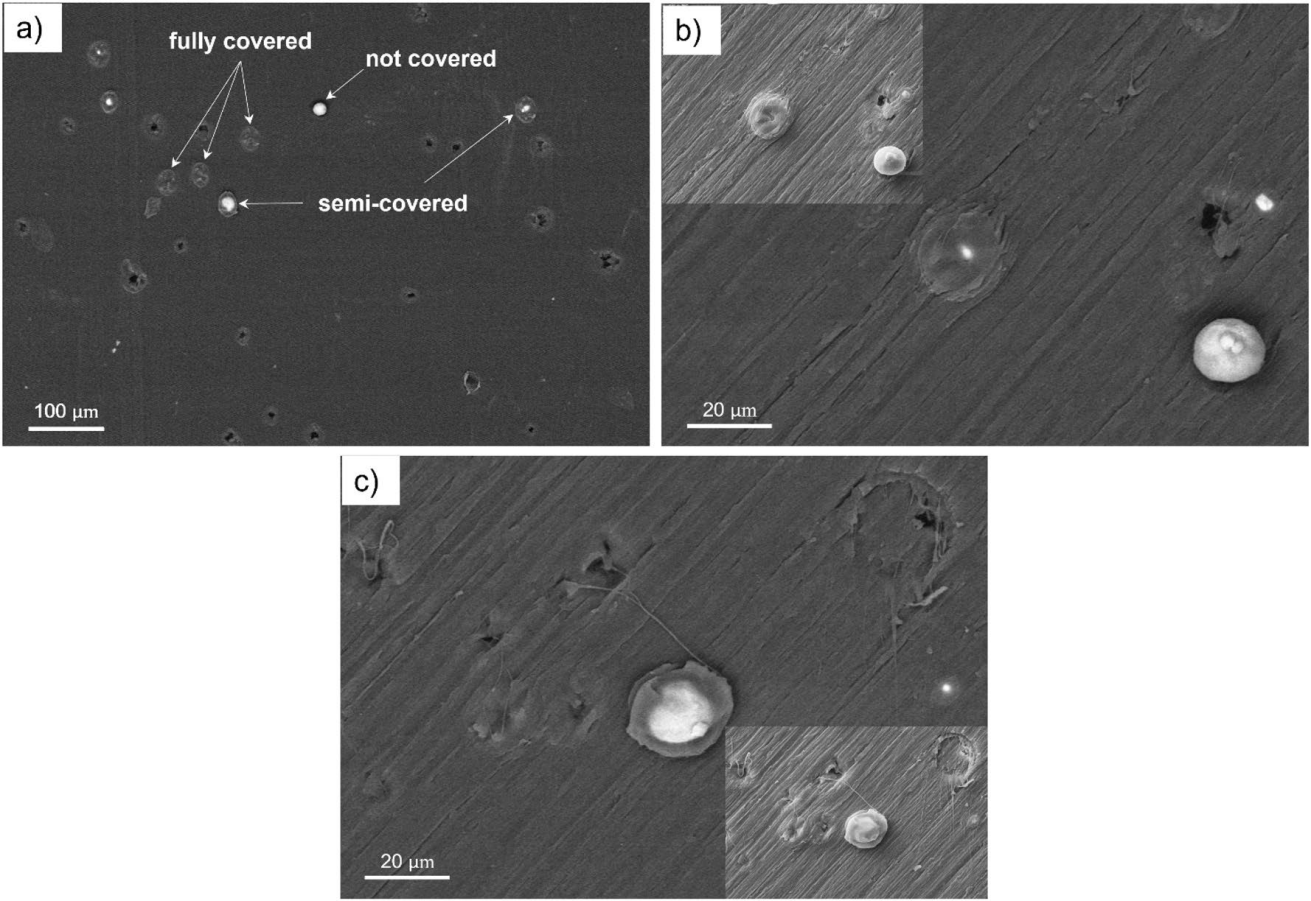
SEM illustration (BSE micrographs) of the PEEK substrate surface after a single-particle impact experiment with $V_{p} = 498 \;{\text{m}}/{\text{s}}$ and ${T}_{p} =101\;^\circ C$: (**a**) at low magnification (**b**) at high magnification depicting fully cov**e**red and not covered and (**c**) semi-covered particles (the inserts are SE micrographs).

Figure 4 depicts top view SEM micrographs of the substrate surface after wipe-test. Figure 4a shows a typical overview of the surface using backscattered electron (BSE) diffraction. The generated impacts appear to be sufficiently far from each other so that the concept of “single-particle” is truly achieved. Due to the heavier nature of elemental copper with respect to PEEK material, Cu particles appear in white in the BSE images in a dark PEEK matrix. The indents without Cu particles are empty craters left after particle detachment. Totally three forms of Cu bonded particles are observed on the surface, as depicted in Fig. 4a indicating various situations that may arise during particle embedment; that is i) some particles are fully covered by a layer of polymer while ii) other particles are either covered partially or iii) have almost no top covering. The empty craters were counted and comprised around 60% of the whole impacted particles based on a total of 219 impacts. This shows that the used conditions in the wipe-test are not favorable for the successful embedment of some particles. This can be considered as a first indicator of the low deposition efficiency usually reported for polymer metallization^37^. This is most probably due to the relatively high kinetic energy of the rebounding particle, which is able to destroy any potential mechanical bond through the deformation and failure of the substrate. Figure 4b,c show a closer view of the embedded and detached particles. In the case of detached particles (Fig. 4c), traces of melted polymer are observed, which indicates that during impact and deformation, a superficial melting may have occurred. In addition, shallow craters indicate a situation of a low energy impact or a harder substrate in terms of lower substrate temperatures which will be discussed later.

**Figure 5:**
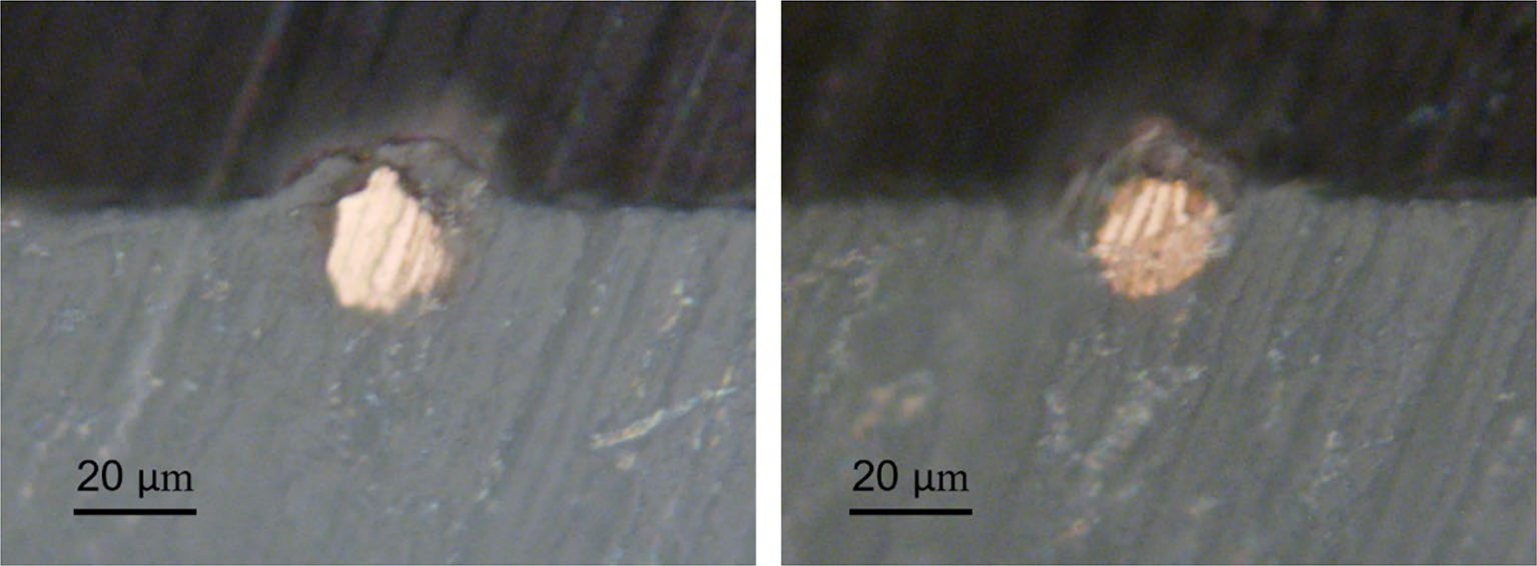
OM images showing the cross-section of two representative interlocked particles.

Figure 5 shows the cross-sections of two representative bonded particles. Due to the hard nature of metallic particles and the soft nature of polymeric substrate, the particles do not exhibit a substantial plastic deformation and maintain their original round shape The polymeric layer seems to be axisymmetrically covering the particle’s perimeter, rising to the upper pole of the particle during impact and subsequent deformation of the substrate. Analyzing the bonded particles with sizes close to the average particle size from the cross-sectional view indicates that the particle penetration depth is generally a fraction of the particle diameter. In other words, such bonded average-sized particles stay on the surface rather than penetrating deeply into the polymer. This is particularly due to the selected cold spray parameter set, which does not permit a drastic deformation in the substrate and thus does not induce a deep penetration of the average-sized particles.

### Numerical simulations

#### Effect of substrate temperature gradient based on the original material model

In this sub-section, the results of the numerical simulation are presented using the original material model used in our previous study (Table 3). Moreover, the effect of the transient substrate temperature gradient on the induced deformations is investigated.

**Table 3:** Exposure time as a function of nozzle speed for typical coating deposition and single-particle impact tests with a flow spot diameter of 8 mm.

Nozzle traverse speed (mm/s)	Exposure time (s)	Test type
50	0.160	Coating deposition
150	0.053	Coating deposition
700	0.011	Wipe-test
1000	0.008	Wipe-test

**Figure 6:**
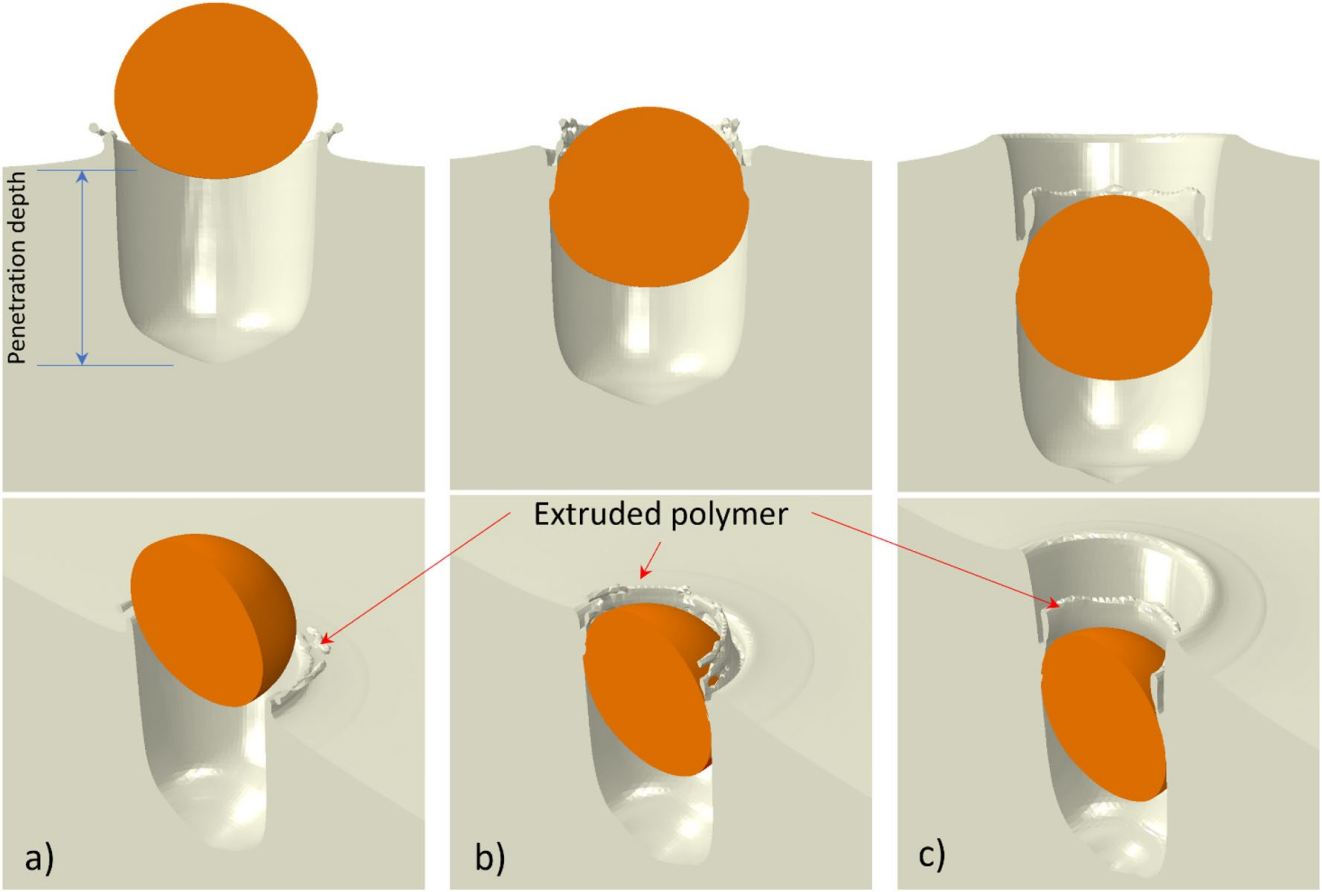
Front and isometric views of the final configuration (with an analysis duration of $5\times {10}^{-7}s$) for particle and deformed substrate using the original material model impacting at ${V}_{p}=500 \mathrm{m}/\mathrm{s}$, ${T}_{p}=100\;^\circ C$: (**a**) ${T}_{s}=25^\circ C$, (**b**) gradient ${T}_{s}$ with ${T}_{s}^{u}=100^\circ C, {t}_{e}=5\times {10}^{-3}s$ (**c**) gradient ${T}_{s}$ with ${T}_{s}^{u}=200^\circ C, {t}_{e}=5\times {10}^{-3}\mathrm{s}$.

Figure 6 shows the results of numerical simulation using $V_{p} = 500\;{\text{ m}}/{\text{s}}$ and $T_{p} = 100\;{ }^\circ {\text{C}},$ which are similar to the average particle velocity and temperature set during the single-particle deposition experiments. Here a final configuration of the particle and substrate is shown after a prolonged analysis duration (5E-7 s). The simulation results are mirrored over a symmetry plane for a better visual perception. Figure 6a shows the case of no substrate heating (i.e., substrate at room temperature) in which the particle generates substantial deformation in the PEEK substrate, forming a crater with a depth of $19.5{ \upmu \text{m}},$ which is as large as the particle size, and then detaching from it when rebounding. The onset of rebound occurs when the particle reaches its maximum penetration depth and the velocity vector of the particle changes direction. However, this model does not seem to match the experimental observations (“Single-particle impact test” section) regarding the interlocked particles. It was shown in our previous work^24^ that for the same particle material and size ($20{ }\;{\upmu m}$), by increasing the particle velocity beyond 550 m/s, bonding was achieved. However, the penetration depth also increased, and a large gap was left under the interlocked particle without any evidence of covering by polymeric layer. Considering these discrepancies with experimental observations, here we updated the model to better match the experimental observations.

To enhance the capacities of the model, the effect of the induced superficial temperature gradient captured by thermography measurements was considered the first candidate for model improvement. An average temperature gradient corresponding to an average exposure time of $5 \times 10^{ - 3} \;{\text{s}}$ with a maximum surface temperature of $100{ }\;^\circ {\text{C}}$ was selected as the base temperature distribution. With such a superficial temperature gradient imposed on the substrate, the crater created by particle impact was even deeper ($24.5{ }\;{\upmu m}$) with respect to the model with no substrate heating (Fig. 6b). This originates from an increase in the deformability of the PEEK due to the imposed temperatures being higher than the room temperature. In this case, when the particle is in the rebound phase, the polymer substrate is extruded outward around the crater, very similar to the effect observed at higher particle velocities^24^. The particle did not leave the crater at prolonged times, stayed bonded to the substrate and the particle velocity vector diminished to zero. The growth of extrusion seems adequate for particle deceleration and thus bonding. To highlight the effect of the thermal domain, another analysis was performed considering an extreme temperature gradient with a maximum surface temperature of $200{ }\;^\circ {\text{C}}$ and an average exposure time of $5 \times 10^{ - 3} \;{\text{s}}$. As observed in Fig. 6a,c much deeper crater was produced ($32.2\;{\upmu {\text{m}}}$), and in this particular case, the particle did not detach and stayed bonded to the substrate due to the thick extruded polymer layer, presumably created as a result of the longer travel distance while rebounding.

Although the two later conditions with temperature gradient exhibit apparent mechanical interlocking, the excessive crater depth, as well as no evidence of covering polymer (in contrast to the observations in “Single-particle impact test” section), indicate a notable mismatch with the experimental results. In addition, the embedded particle in the numerical model is accompanied by a gap below it, which does not seem to be a general phenomenon in the experiments. In summary, it can be concluded that the addition of a superficial thermal domain did not result in a significant enhancement in the prediction of the original model. Examining the obtained results indicated that the PEEK material did not demonstrate a correct behavior, failing to capture the observed phenomena in the single-particle impact experiments, and thus it required further attention.

#### Effect of material model improvement together with temperature gradient

**Figure 7:**
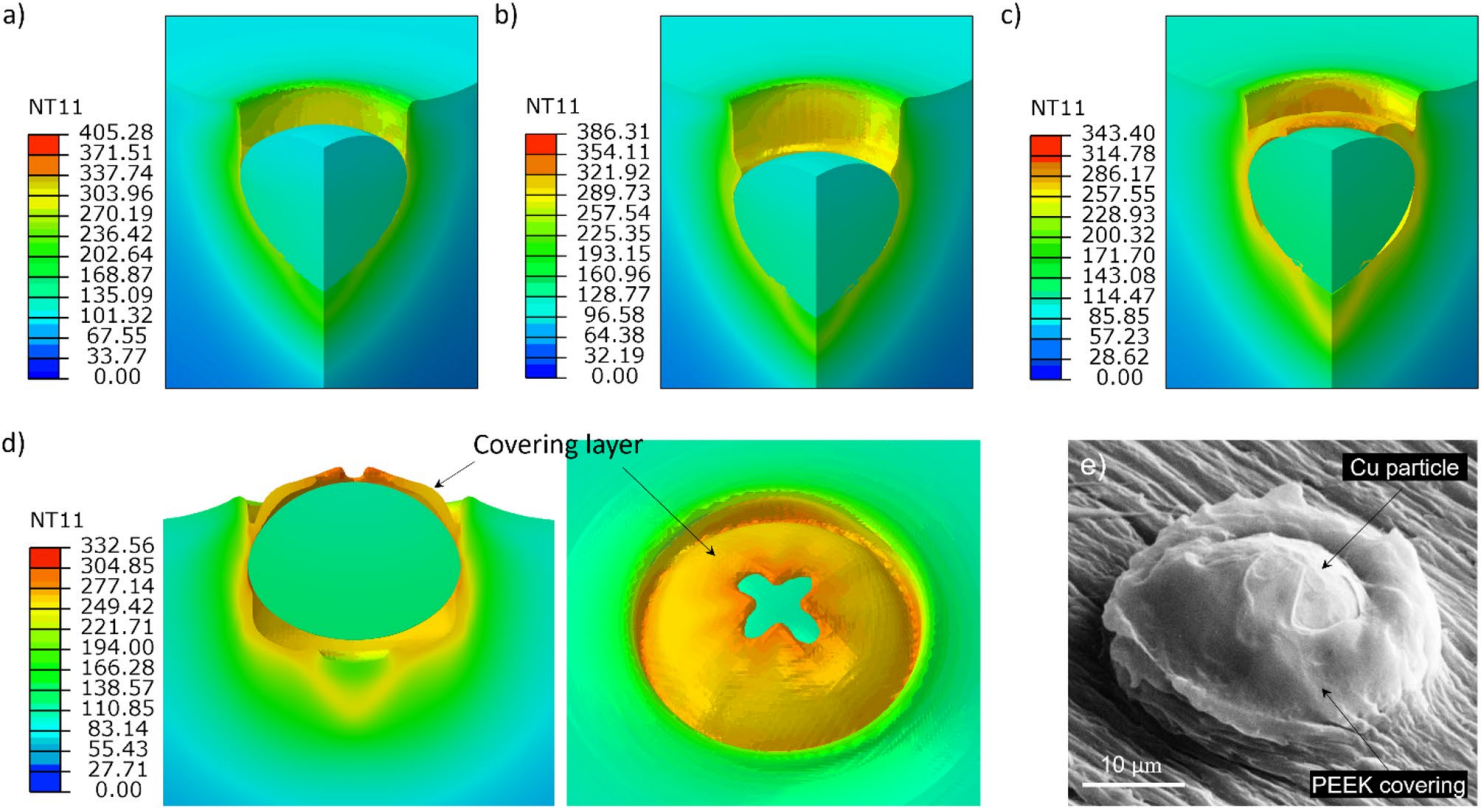
Deformation features and temperature field of the Cu particle and the PEEK substrate using improved material hardening/softening behavior, impacting at ${V}_{p}=500\; \mathrm{m}/\mathrm{s}$; the snapshots correspond to impact times of (**a**) $4 \times 10^{ - 8}\; {\text{s}}$ (**b**) $6 \times 10^{ - 8} \;{\text{s}}$ (**c**) $11 \times 10^{ - 8}\; {\text{s}}$ and (**d**) $13 \times 10^{ - 8}\; {\text{s}}$; (**e**) SEM illustration of a bonded particle with a partially covered PEEK layer.

The effects of the modified PEEK material model in terms of hardening/softening behavior are illustrated in Fig. 7. Overall deformation features including particle penetration into the substrate, intermediate stages of substrate deformation, and particle rebound are similar to the ones introduced in our previous numerical study^24^. Therefore, the deformation details due to the modified material model are described below.

Figure 7 shows the deformation stages of a single-particle impact modeled using the modified material model. The model incorporates also an average temperature gradient of $T_{s}^{u} = 100\; ^\circ {\text{C}}, \;t_{e} = 5 \times 10^{ - 3} \;{\text{s}}$. In this figure, the times listed in the caption indicate the interval after the initiation of the impact. During the particle impact, due to the increased deformability of the PEEK at higher temperatures, a layer of material starts to be extruded out close to the interface of the particle and the crater, even before the onset of particle rebound (Fig. 7a,b). With the continuing deformation, this layer grows outside of the substrate surface and partially covers the upper surface of the particle (Fig. 7c). A 3D perspective is also provided in Fig. 7d at a later stage showing the developed covering layer. A Cu particle bonded to the PEEK substrate with a partial covering layer is shown in Fig. 7e from a closer view. Comparing Fig. 7d,e, indicates that with the improved model (modifying material behavior and also considering a superficial temperature gradient), the formation of a covering layer on the particle can be simulated successfully. With the continuation of the analysis time, however, it is found that the particle rebounds and eventually detaches from the substrate. The particle’s kinetic energy is so large during the rebound that the covering layer is torn off and the particle loses its bond with the substrate.

The results showed that the deformation of PEEK in correspondence to the crater depth is lowered ($14\; {\upmu {\rm{m}}}$) in comparison to the original model with the same temperature gradient field (Fig. 6b). This originates from the enhancement in material hardening, especially at low temperatures. However, by adding the softening behavior at temperatures close to the melting point, the extrusion of the covering polymer was modeled correctly.

Because the covering layer in the numerical model is not able to retain the particle in place and prevent the detachment, it is logical to think of another possible contributing factor for a successful bonding of the particles. This aspect becomes more important to model also the case of those interlocked particles, which turn out to have almost no covering layer on the top (Fig. 4a). It is postulated that this could be, to some extent, caused by the continuous high-speed flow of the propellent gas during deposition. The extruded polymer layer that partially covered the particle perimeter is softened due to high temperatures (as evident in Fig. 4b,c) and thus can be easily deformed under the continuous expanding gas flow. For the cold spray processing parameters utilized in this study, a gas velocity of $50\; {\text{m}}/{\text{s}}$ and a gas pressure of $0.7\; {\text{MPa}}$ are predicted by the KSS software in the zone very close to the substrate surface. This gas flow can deform the “hot polymer” making a barrier for the particle rebound and hindering it from detaching. Thus, the flattened and squeezed covering layer in Fig. 4c could be induced by the gas flow effect.

**Figure 8:**
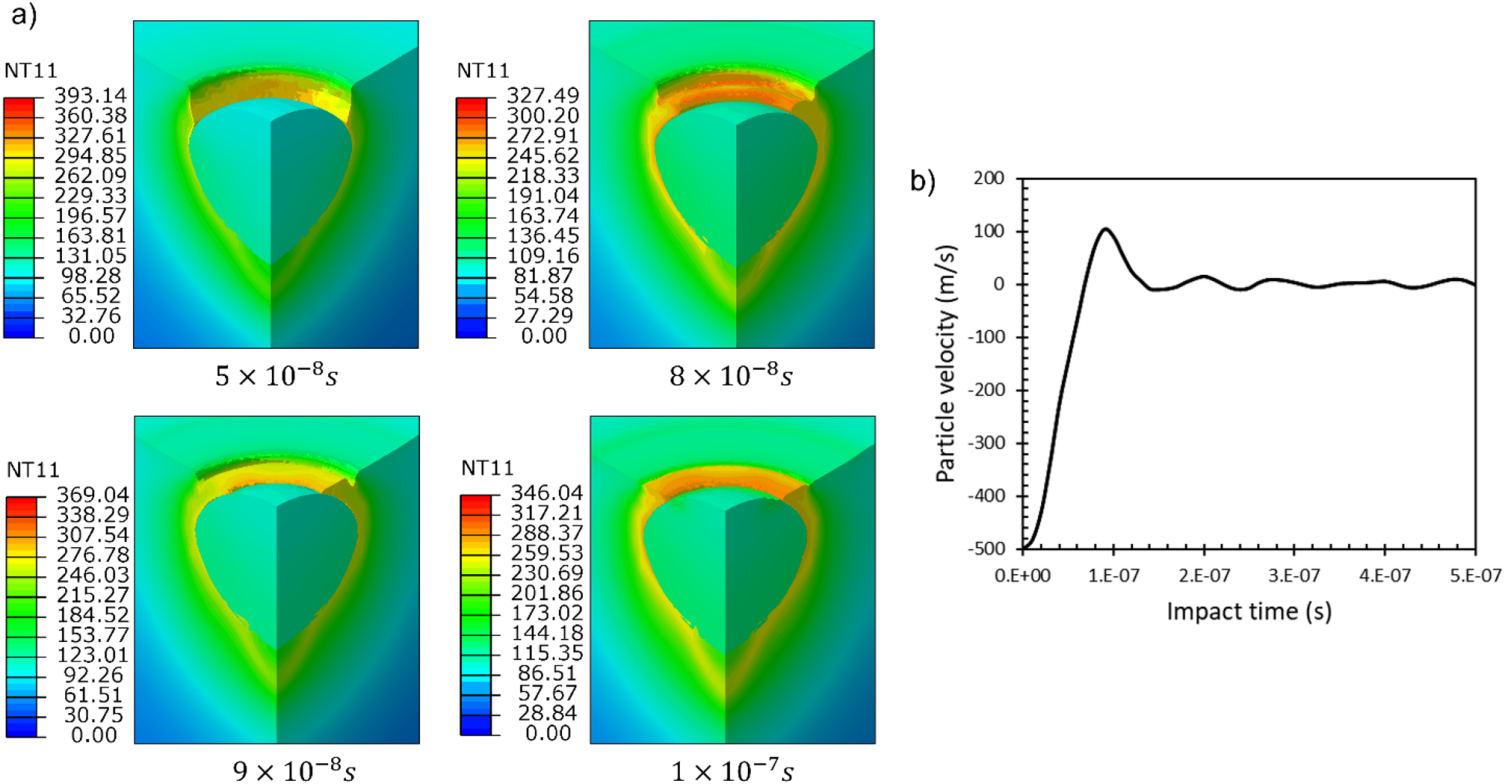
(**a**) Deformation stages of the substrate and particle during the impact with ${V}_{p}=500\; \mathrm{m}/\mathrm{s}$ after addition of the gas flow compensating effect (**b**) particle velocity variation as a function of impact time.

To model such a contribution from the gas flow, an infinitesimal velocity boundary condition (${V}_{y}=-1\times {10}^{-15}$ $\mathrm{m}/\mathrm{s}$ )was applied in the vertical direction to all the Eulerian elements located higher than the initial surface level in the center within a radius 2 times that of the particle. Initially, this region is without any material, representing an Eulerian void. This boundary condition was applied to the deforming PEEK as it flowed into this region. The results of the simulation are shown in Fig. 8. At the time interval of $5 \times 10^{ - 8} \;{\text{s}}$, the particle achieved its maximum penetration and started rebounding. It is observed that the extrusion of the PEEK layer already started at the interface of the particle and the crater. Since the PEEK material was extremely softened, it flowed and filled the gap between the extruded layer and the surrounding polymer (at $8 \times 10^{ - 8} \;{\text{s}}$ and $9 \times 10^{ - 8} \;{\text{s}}$). At $1 \times 10^{ - 7} \;{\text{s}}$, the gap was filled, and the deformation stopped due to the counterbalancing effect from the gas flow. The particle’s velocity reduced to zero at longer analysis times (Fig. 8b). This analysis shows an interlocked particle with a very small partial covering layer over the particle perimeter and no excess gap below the particle, similar to what was observed in our experimental wipe-tests (Fig. 5). The addition of the gas flow effect also removed the creation of any bulge out of the initial surface in the surrounding substrate, which lowers the final penetration depth, making it closer to the experimental observations (Fig. 6).

## Discussion

A numerical FE model was developed to simulate metallic particle impact on a polymeric substrate deposited using cold spray technology. This study is an advancement with respect to the authors’ previous work that suggested a preliminary model for the cold spray metallization of polymers. This new model is validated by comparison with experiments of wipe-test (single-particle impact).

By examining the deposited single particles and the relevant features in the wipe-test, various particle–substrate interaction states were identified (Fig. 4). For bonded particles, the penetration depth varied between half the particle diameter and its full diameter. The not interlocked particles left an empty crater with a certain depth and size over the surface. Some rationalities could be hypothesized for the creation of such diverse features. Particle velocity variation could be the main factor as the in-flight particle velocity in cold spray can vary due to shape irregularities^38,39^. Furthermore, particle velocity and temperature variations in practice are expected with respect to CFD simulations. The temperature history of the substrate during the spray can also be other potential explanations for the formation of different impact features. These irregularities may have caused relatively low deposition efficiency in the single-particle impact test (“Single-particle impact test” section).

**Figure 9:**
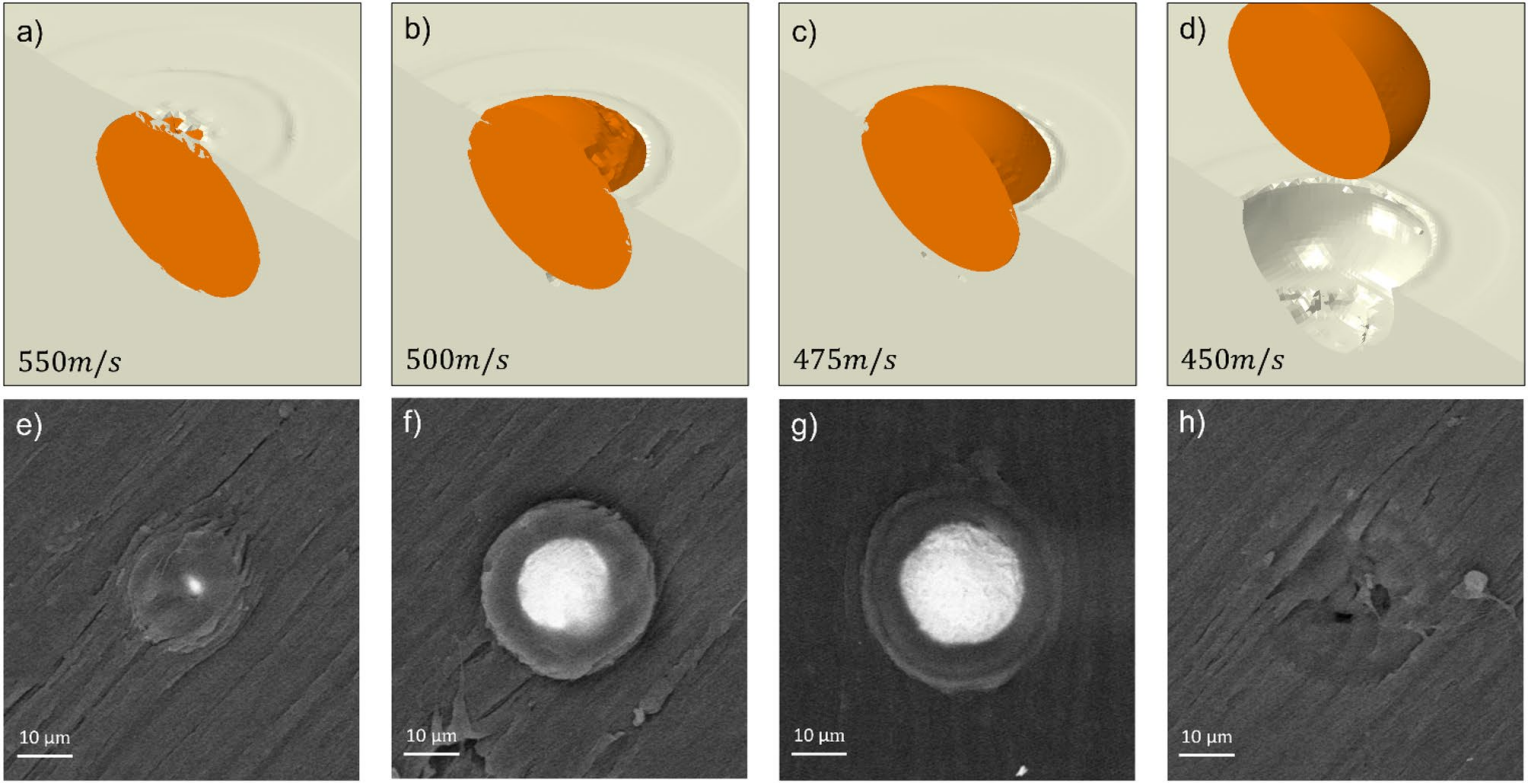
The effect of particle velocity fluctuations on the extent of particle bonding (**a**) $550\; \mathrm{m}/\mathrm{s}$ (**b**) $500\; \mathrm{m}/\mathrm{s}$ (**c**) $475\; \mathrm{m}/\mathrm{s}$ (**d**) $450\; \mathrm{m}/\mathrm{s}$ (**e**–**h**) experimental features similar to a–d, respectively.

As the first candidate, the effect of variation in the particle velocity ($\pm 50 \;{\text{m}}/{\text{s}}$) on the particle interlocking was investigated using the proposed model, the results of which are reported in Fig. 9. It can be seen that an increase in the velocity by $50 \;{\text{m}}/{\text{s}}$ beyond the mean value resulted in a bonded particle with an almost fully covered layer on top (Fig. 9a). This configuration is produced as a result of increased penetration depth due to higher kinetic energy of the particle. As the velocity was decreased below $500 \;{\text{m}}/{\text{s}}$ (Fig. 9b) by $50\; {\text{m}}/{\text{s}}$, the penetration depth reduced and around half of the particle height remained uncovered outside the surface (Fig. 9c). By further decrease in velocity (450 ${\text{m}}/{\text{s}}$), there was not sufficient penetration of the particle and thus, no mechanical interlocking was achieved, instead a crater was left at the impact location (Fig. 9d). Figure 9e–h illustrate the experimental findings over the single-particle impact tests, which reflect, in order, features similar to the observations in the numerical simulations within the examined particle velocity range.

**Figure 10:**
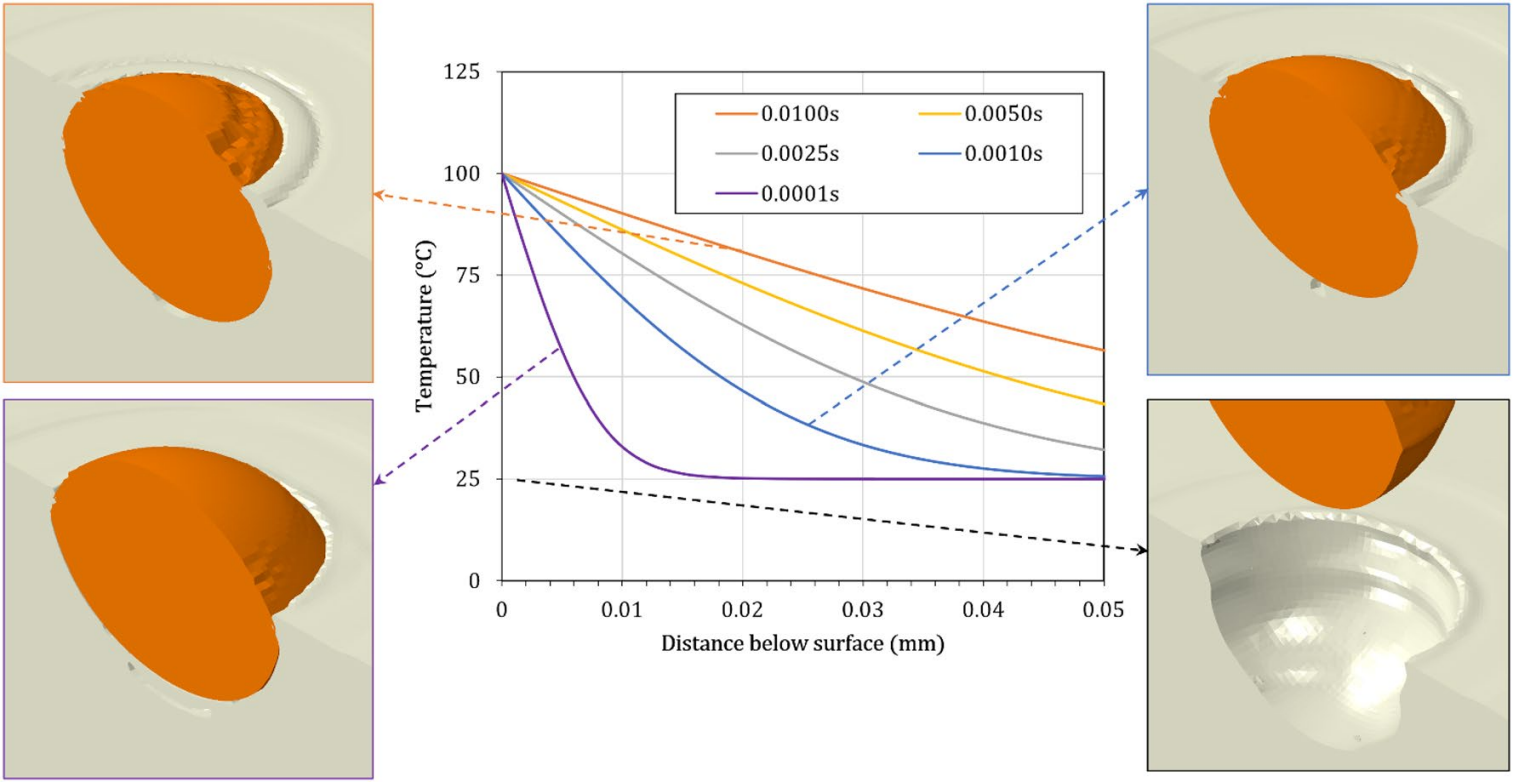
The effect of substrate temperature gradient on particle bonding at ${V}_{p}=500\; \mathrm{m}/\mathrm{s}$ at a sufficiently prolonged time. Times listed in the legend of the diagram, indicate the time that particle arrives at the substrate during exposure.

As the second probable source of distinct configurations, the varying temperature gradient in the substrate was emphasized. Figure 10 shows the effect of the temperature gradient on the bonding phenomenon at the mean particle velocity of $V_{p} = 500 \;{\text{m}}/{\text{s}}$. As the time increased, the gradient levelled off, and higher temperatures were imposed through the substrate depth. Thus, the particle penetration depth increased. This is in line with available reports for various polymers where the penetration depth increased with the increase of the gas temperature^21^. A limiting case of a very steep gradient can be imagined at the very start of spray exposure such that the surface layer can be assumed to be momentaneously at room temperature. In this case, as depicted on the bottom right in Fig. 10, the particle loses its bond and detaches from the substrate due to a limited deformation in the substrate. This implies that the modified material model is not able to catch the bonding phenomena without imposing a temperature gradient. Therefore, both the modified material model and the gradient thermal domain seem to be important for the correct simulation of particle bonding.

As the last step to complete the model, the effect of gas flow was suggested as a player in the successful bonding of the particle. Surely the growth of the covering polymer layer on the particle can be simulated more realistically with a more sophisticated model considering the real gas flow through a fluid dynamics model. However, here we selected a more convenient way to simulate the effect of gas pressure by applying a velocity boundary condition as an initial step to overcome the complexities.

The effect of nozzle traverse speed on the quality of cold spray coatings on metal substrates has been investigated intensively. A faster nozzle speed usually leads to lower substrate temperatures, which in turn hinders the severe deformation in particles, producing a more porous coating^40,41^. Hardness, adhesion strength, and deposition efficiency are also reported to be influenced by nozzle traverse speed^40–44^. Nevertheless, as regards cold spraying on polymeric substrates, the authors did not find any available studies on the effect of nozzle traverse speed. However, the results of the current study suggest the nozzle traverse speed to be a controlling parameter for the quality of the coatings produced on polymeric substrates. An interesting outcome could be the effect of overall penetration depth and interface nature on the adhesion strength of the coating. It could be hypothesized that the penetration depth would change with different nozzle speeds, resulting in a distinct interlocking and adhesive strength.

**Figure 11:**
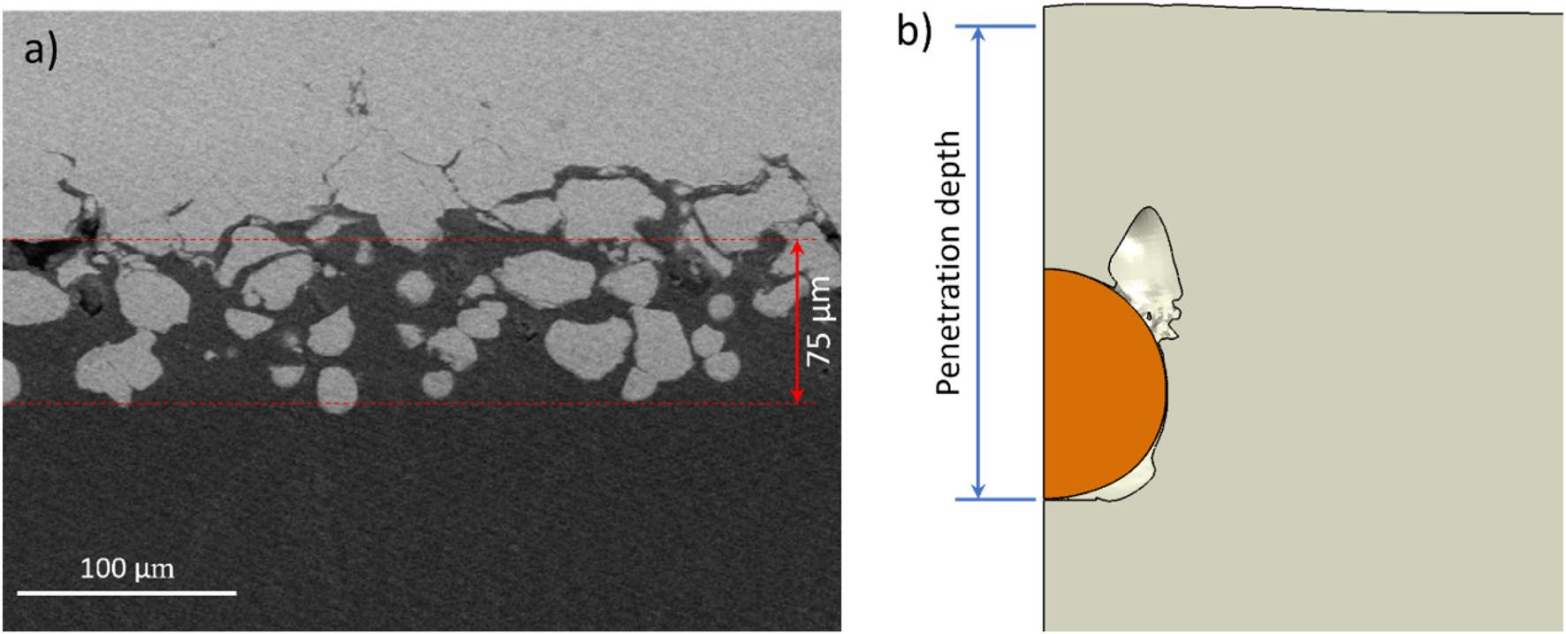
(**a**) Cross-ional view of the interface for Cu coating and PEEK substrate deposited using cold spray parameters listed in Table 1 (**b**) numerical simulation of a single Cu particle impact on PEEK with a constant substrate temperature of 200 °C at 4.0×10^−7^s.

Based on the above discussion, it can be deduced that the induced temperature gradient on the substrate is enhanced during coating build-up in comparison to the single-particle impact deposition, due to lower nozzle traverse speeds and a larger number of simultaneous impacts. Accordingly, the layer immediately below the surface experiences very high temperatures with a higher depth of thermal diffusion. In addition, one must consider the varying temperature gradient as a function of time during the nozzle passage (Fig. 10). Figure 11a shows the experimental coating interface between the deposited Cu layer and the PEEK substrate using the parameters listed in Table 1. The coating was deposited using a nozzle traverse velocity of $30 \;{\text{mm}}/{\text{s}}$. A diffusive bond layer or interfacially mixed layer was formed, which is also reported in other Cu/PEEK cold spray coatings^8,19^. Considering such low nozzle traverse velocity, the induced superficial temperature would be around $200\;{ }^\circ {\text{C}}$ (experimentally measured as in Fig. 3b) and would remain almost constant through a depth of $100\;{\upmu {\rm{m}}}$ according to the numerical simulation (as shown in Fig. 2b). This can be considered as the reason behind those particles that appear much deeper at the coating interface (Fig. 11a). To examine this hypothesis, a numerical analysis was done using a $200 \;^\circ {\text{C}}$ constant temperature field through the substrate domain corresponding to a nozzle velocity of $30\; {\text{mm}}/{\text{s}}$. The penetration depth was found to be $\sim 40 {\upmu {\rm{m}}},$ which is twice the particle size. The thickness of the overall diffusive layer in the interface was measured to be $\sim 75 \;{\upmu {\rm{m}}}$. The initial undeformed surface was noted to be displaced due to particle embedment and coating build-up. The results indicate that the final penetration depth of particles depends on their velocity, which is affected by their size and shape, and the substrate exposure time to the gas flow. However, the modeled penetration depth ($40{ \upmu \text{m}}$) for the average particle size and velocity is of the same order of magnitude compared to the measured thickness ($75 {\upmu {\rm{m}}}$ of the diffusive layer (Fig. 11). It should be kept in mind that smaller particles with higher velocities may penetrate deeper and result in a larger diffusive layer. Additionally, there could be multiple successive impacts in the experiments that may potentially induce a peening effect for pushing further the particles into the substrate. The polymer may also soften substantially at temperatures higher than the glass transition point, which contributes to increasing the penetration depth.

The nature of the interface has been proved to affect the coating adhesion strength^45^. The formation of the diffusive bond layer at the interface can be an important factor in determining the coating adhesion. Thus, it is deduced that apart from the main processing parameters, i.e., the gas temperature and pressure, also the nozzle traverse speed can render a certain effect on adhesion strength. This could be investigated in a future experimental study by adjusting the feed rate such that the coating thickness remains constant as the nozzle traverse speed changes.

## Conclusions

A numerical finite element model was developed to simulate the bonding of metallic particle impact on the thermoplastic polymers by cold spray deposition. Experimental cold spray deposition of copper powder feedstock was performed on a polyether ether ketone (PEEK) substrate to provide realistic data for model construction as well as its validation. The following conclusions are drawn:

- Due to exposure to the gas stream, the substrate heats up temporarily with a temperature gradient in the surface layer within a duration of several nanoseconds. The maximum temperature being on the topmost surface depends strongly on the nozzle traverse speed. The temperature gradient, which is varied during the exposure time, seems to influence largely the deformation behavior of PEEK and the subsequent particle bonding.
- The implemented material model for the PEEK substrate accounting for the high strain rates and high temperatures encountered in cold spray was successfully validated using the experimental single-particle impact tests. Proper calibration of PEEK material hardening behavior, as well as its softening at sufficiently high temperatures, together with the consideration of transient substrate heating during gas stream exposure, considerably enhanced the agreement between numerical model and experimental observations.
- The proposed model could predict various particle impact features on the polymer by taking into account the particle velocity variation and substrate temperature gradient. The model successfully simulated particle interlocking, proper deformation of the substrate with no gap below the particle, the covering layer on the embedded particle, and its rebound leaving an empty crater.
- Based on the simulation outcomes, it is concluded that for an effective interlocking, the gas stream is also engaged as a contributing factor in particle deceleration during rebound and consequently, particle bonding.
- To sum up, this work elaborates on major difficulties in developing numerical models for the simulation of metal particle impact and bonding to thermoplastics. It established the first steps in building a reliable numerical model that reflects realistic impacts and bonding phenomena in thermoplastic polymer metallization. The developed model can be further expanded to be used to study the adhesion strength of single particles and correlate it to the one of the coatings in order to provide a numerical scheme for its estimation. Furthermore, this model can be used as a basis in future works to develop numerical models and simulate the cold spray deposition of multiple-particle impacts. This in turn enables the optimization of the process parameters for polymer metallization with favorable deposition efficiency.

## Supplementary Information

Supplementary Information.

## Data Availability

The datasets used and/or analysed during the current study are available from the corresponding author on reasonable request.
