# Supplementary material for: Insights on metallic particle bonding to thermoplastic polymeric substrates during cold spray
Source: Sci Rep. 2022 Oct 27;12:18123. doi: 10.1038/s41598-022-22200-5 (PMC9613992; doi:10.1038/s41598-022-22200-5)
Supplement: Supplementary file 1 — Supplementary Information. [file 41598_2022_22200_MOESM1_ESM.docx]

# Supplementary Data


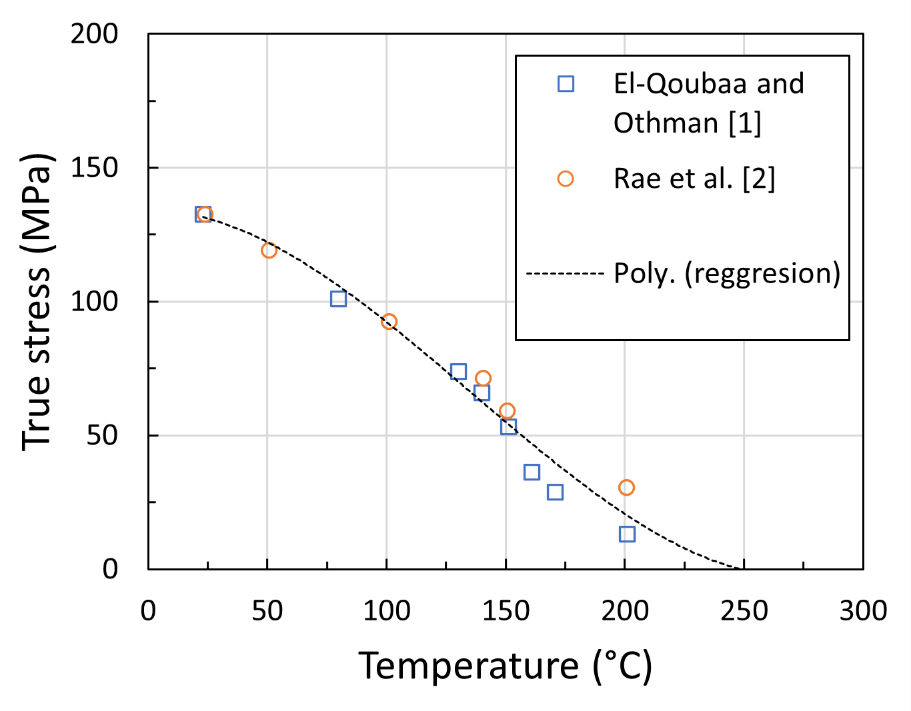


Fig. S1 Extrapolation of experimental yield stress data of PEEK as a function of temperature to zero stress

[1] El-Qoubaa, Z. & Othman, R. Temperature, Strain Rate and Pressure Sensitivity of the Polyetheretherketone’s Yield Stress. *Int. J. Appl. Mech.* **09**, 1750099 (2017).

[2] Rae, P. J., Brown, E. N. & Orler, E. B. The mechanical properties of poly(ether-ether-ketone) (PEEK) with emphasis on the large compressive strain response. *Polymer* **48**, 598-615 (2007).
